# Sequence and structural features of carbohydrate binding in proteins and assessment of predictability using a neural network

**DOI:** 10.1186/1472-6807-7-1

**Published:** 2007-01-03

**Authors:** Adeel Malik, Shandar Ahmad

**Affiliations:** 1 Department of Biosciences, Jamia Millia Islamia University, New Delhi-110025, India

## Abstract

**Background:**

Protein-Carbohydrate interactions are crucial in many biological processes with implications to drug targeting and gene expression. Nature of protein-carbohydrate interactions may be studied at individual residue level by analyzing local sequence and structure environments in binding regions in comparison to non-binding regions, which provide an inherent control for such analyses. With an ultimate aim of predicting binding sites from sequence and structure, overall statistics of binding regions needs to be compiled. Sequence-based predictions of binding sites have been successfully applied to DNA-binding proteins in our earlier works. We aim to apply similar analysis to carbohydrate binding proteins. However, due to a relatively much smaller region of proteins taking part in such interactions, the methodology and results are significantly different. A comparison of protein-carbohydrate complexes has also been made with other protein-ligand complexes.

**Results:**

We have compiled statistics of amino acid compositions in binding versus non-binding regions- general as well as in each different secondary structure conformation. Binding propensities of each of the 20 residue types and their structure features such as solvent accessibility, packing density and secondary structure have been calculated to assess their predisposition to carbohydrate interactions. Finally, evolutionary profiles of amino acid sequences have been used to predict binding sites using a neural network. Another set of neural networks was trained using information from single sequences and the prediction performance from the evolutionary profiles and single sequences were compared. Best of the neural network based prediction could achieve an 87% sensitivity of prediction at 23% specificity for all carbohydrate-binding sites, using evolutionary information. Single sequences gave 68% sensitivity and 55% specificity for the same data set. Sensitivity and specificity for a limited galactose binding data set were obtained as 63% and 79% respectively for evolutionary information and 62% and 68% sensitivity and specificity for single sequences. Propensity and other sequence and structural features of carbohydrate binding sites have also been compared with our similar extensive studies on DNA-binding proteins and also with protein-ligand complexes.

**Conclusion:**

Carbohydrates typically show a preference to bind aromatic residues and most prominently tryptophan. Higher exposed surface area of binding sites indicates a role of hydrophobic interactions. Neural networks give a moderate success of prediction, which is expected to improve when structures of more protein-carbohydrate complexes become available in future.

## Background

**Figure 1:**
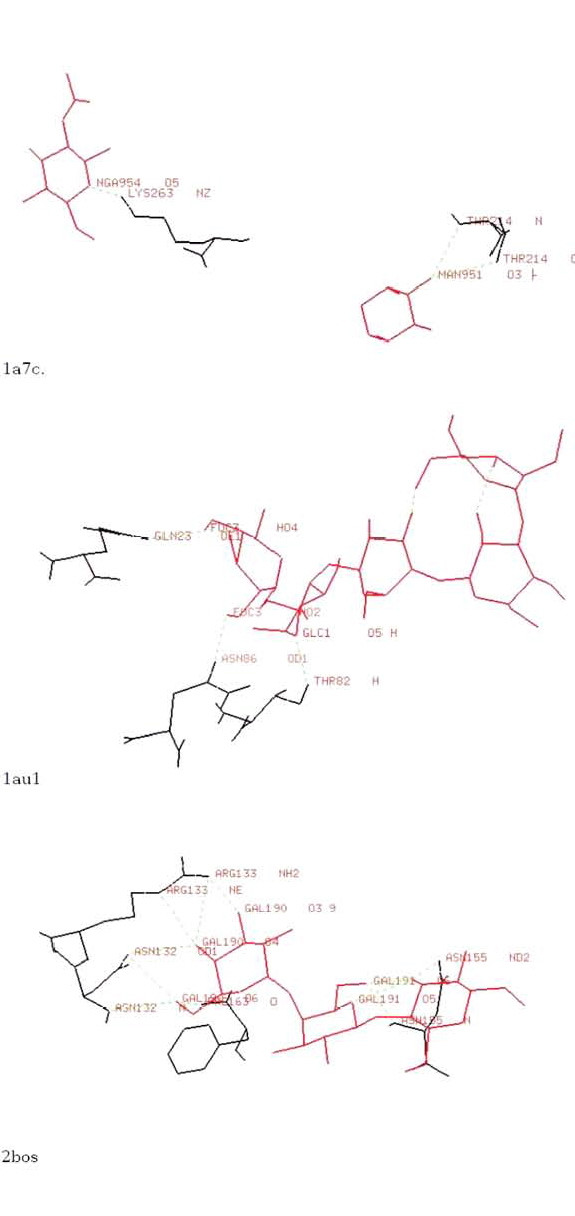
Some typical protein-carbohydrate interactions. All the atoms making hydrogen bonded contacts between sugars and amino acids are labelled.

Carbohydrates are often referred to as the third molecular chain of life, after DNA and proteins [1]. These interactions are responsible for important biological functions such as inter cellular communication particularly in the immune system [2]. Living cells in all organisms are usually covered with one or another type of carbohydrate [2]. Some viruses like influenza, use sugars on the outside of human cells to gain entry. Sometimes the carbohydrate-binding proteins and their sugar-ligands are expressed on the same cell, and the sugar is a part of the regulation machinery of the cell [3]. The functional roles of carbohydrates and their interactions with proteins are drawing more attention than before, since it has been recognized that carbohydrates are used as information carriers, rather than simple storage material [1]. Protein-carbohydrate interactions are involved in a broad range of biological processes. These processes include, among others, infection by invading microorganisms and the subsequent immune response, leukocytic trafficking and infiltration, and tumor metastasis [4-12]. Carbohydrates are uniquely suited for this role in molecular recognition, as they possess the capacity to generate an array of structurally diverse moieties from a relatively small number of monosaccharide units [13]. This could be attributed to the fact, that unlike the components of nucleic acids, carbohydrates can link together in multiple, nonlinear ways because each building block has about four functional groups for linkage. They can even form branched chains. Hence, the number of possible polysaccharides is enormous (Figure 1). Since carbohydrates assume a large variety of configurations, many carbohydrate-binding proteins are being considered as targets for new medicines.

In view of the above, accurate *in silico *identification of carbohydrate-binding sites is a key issue in genome annotation and drug targeting. The information about the factors, which prevent or support carbohydrate binding of an amino acid, is expected to be present in the evolutionary profile of the sequence as well as the identity and structure of amino acid residues in the neighborhood of potential carbohydrate binding sites. A number of reviews have been published on protein- carbohydrate interactions [14-18]. Different aspects of protein carbohydrate recognition have also been extensively studied [19-26]. However, bioinformatics approaches with a predictive goal are relatively rare [1,27]. Compared with the abundance of methodologies developed for protein-nucleic acid [28-32] or protein-protein interactions [33-38], there are still very few methods for predicting carbohydrate-protein interactions. Shionyu-Mitsuyama [1] has developed a program that uses the empirical rules of the spatial distribution of protein atoms at known carbohydrate-binding sites for prediction. In that work an analysis of the characteristic properties of sugar binding sites was performed on a set of 19 sugar binding proteins. For each site six parameters were evaluated viz. solvation potential, residue propensity, hydrophobicity, planarity, protrusion and relative accessible surface area. Three of the parameters were found to distinguish the observed sugar binding sites from the other surface patches. These parameters were then used to calculate the probability for a surface patch to be a carbohydrate-binding site [27]. These prediction methods are based on local structural descriptors of proteins and cannot be used if complete 3 dimensional structures are not available. On the other hand, neural network based predictions of post-translational modification (O-glycosylation and phosphorylation) sites have been reported by two groups [39,40]. However, these studies are restricted to only one type of protein-carbohydrate interactions and therefore do not capture all protein-carbohydrate interactions, as sought out in this work.

In this work we explore the exact contribution from different sequence and evolutionary attributes of proteins in determining their carbohydrate binding regions. Propensity of each of the 20 amino acid residues in binding regions has been calculated and compared with non-binding regions. Solvent accessibility, secondary structure and packing density of binding sites have been analyzed in a similar way. We go on to design a neural network to model sequence and evolutionary information (obtained by Position Specific Scoring Matrices) and determine their role in the predictability of carbohydrate binding sites.

We also study the binding sites of other protein-ligand and protein-DNA complexes and compare the propensity scores of all residues and their secondary structures with protein-carbohydrate complexes.

## Results and Discussion

### Residue-wise propensity scores

**Figure 2:**
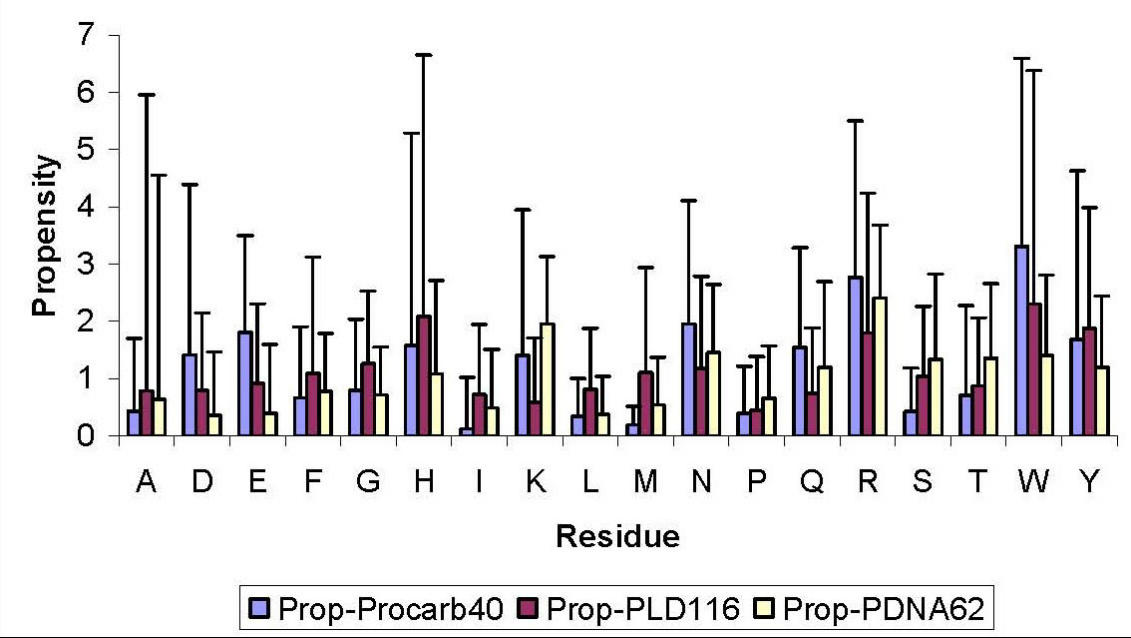
Comparison of binding site propensity of each residue in Procarb40, PDNA62 & PLD116 (residue was marked as binding if any of its atom fell within 3.5 Å of any atom of the ligand/DNA/carbohydrate. Propensity values were obtained by pooling all residues of the same type in all proteins to a single database of binding and non-binding sites. To compute the error bars, propensity values were calculated for each protein separately and standard deviations in propensity values was used as an error bar.

We started with a non-redundant set of all carbohydrate binding proteins (Procarb40) collected from PDB as described in the Methods section. Residue-wise propensities of carbohydrate binding sites in Procarb40 dataset were calculated and compared with the propensities of protein-ligand interaction database PLD116 and protein-DNA interaction database PDNA62 complexes, former of which is used as control data sets and the later for additional comparison. These datasets are described in Methods section. Results obtained from this analysis are summarized in Figure 2 and Table 2. It may be observed that certain residues (e.g. TRP, GLN, and ASN) are over-represented within the binding sites of these 40 protein-carbohydrate complexes, which signifies their importance in protein-carbohydrate interactions. These results may be understood in the light of reported experimental and theoretical studies on carbohydrate interactions. For example, it has been argued that the side chain residues with polar planar groups- ASN, ASP, GLU, GLN, ARG, and HIS- are the only ones participating in all three forms of hydrogen bonding with sugars and are abundant in sugar-binding sites, which explains why their propensities in the binding sites is higher [14]. Our analyses show that aromatic amino acid residues are often present in carbohydrate-binding sites of proteins. These binding sites are characterized by a placement of a carbohydrate moiety in a stacking orientation to an aromatic ring. This arrangement is an example of CH/pi interactions, which have been shown to play an important role in carbohydrate recognition by glycosidases and carbohydrate-binding proteins [41]. Apart from confirming some of the widely accepted ideas on residue preference for carbohydrate binding, our study determines exact role and contribution of each residue to carbohydrate binding.

Highest propensity score (331% over representation) in the carbohydrate binding sites is observed from Figure 2 and Table 2 for tryptophan (TRP), which is in accordance with many reported mutational studies [42]. This and other studies have provided experimental and theoretical evidence that the presence of TRP residues in mutation sites is crucial for their binding to carbohydrates [43-45]. Additionally, the conservation of aromatic residues, such as tyrosine and phenylalanine, on an exposed surface is common in carbohydrate-binding modules (CBMs) from families 1, 3, 5 and 10, highlighting the role of aromatic residues in carbohydrate binding [46-50]. (It may be noted that CBMs have been previously classified into different families in which groupings of Carbohydrate binding domains or CBDs were called "Types" and numbered with roman numerals (e.g. Type I or Type II CBDs) [51]).

The modification of tryptophan residues has also been shown to cause a compete loss of hemagglutinating activity [52]. Involvement of two tryptophan residues in carbohydrate-binding site was also shown to be essential in the same study. Similarly, Lafora disease-related mutation of TRP32 to glycine (W32G) has also been shown to disrupt the polysaccharide-binding pocket and also potentially unfold the region immediately adjacent to the binding pocket [53]. All these experimental results are well reflected in the high propensity of TRP in carbohydrate binding sites presented in this work (Figure 2).

If the propensity scores of carbohydrate binding are compared with other ligand-binding residues identified by PLD116 database (see Methods), TRP remains the most prominent high propensity residue. However, high propensity score of HIS residues is also shown by PLD binding sites, indicating that the role of HIS in ligand-binding sites does not have specific preference for carbohydrates, but HIS in general being an active site shows high propensity of binding to any ligand, including carbohydrates. Next important residue is ARG whose propensity for carbohydrate binding is less than TRP within Procarb40, yet the propensity (277% over representation) is even higher than what is observed for ARG in DNA-binding proteins database PDNA62 (= 241% over representation) (see Table 2 and Methods section). These results are supported by some published results of transmutagenesis experiments reporting crucial role of ARG residues in some protein-carbohydrate interactions [54]. Lower propensity scores for the other basic residue LYS indicate that the interaction between ARG and sugar is not purely electrostatic in nature. Dahms *et al*. in 1993, [54] also report that the substitution of ARG residues by LYS in Insulin-like growth factor also caused loss of binding despite similar electropositive property of these residues and also despite overall conservation of structure upon this mutation. These results were interpreted that the proteins utilize residues with planar side chains (ARG, ASN, ASP, GLU) for their interaction with sugars. Higher propensity scores of ASP and GLU, which are also negatively charged residues, also support this argument. These propensity scores are higher than what is observed for other ligands (PLD116 database), thus highlighting a preference of these residues to interact carbohydrates in contrast to other types of ligands. In comparison to DNA-binding propensity scores of ASP and GLU are much higher, obviously because negatively charged bases in DNA repel negative charged residues.

### Solvent accessibility of binding sites compared with the rest of the protein

**Figure 3:**
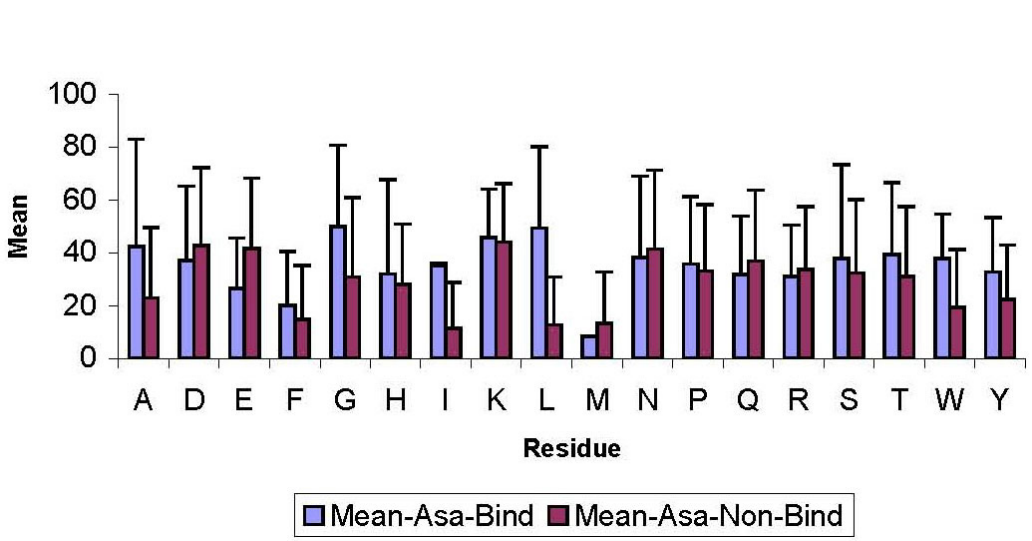
Comparison between mean ASA values of residues in binding and non-binding sites for Procarb40. Error bars are taken from their standard deviation in each protein. The graph does not contain cystein and valine data as none of these residues were found to be in the binding regions.

We next attempted to establish a residue-wise relationship between solvent accessibility and carbohydrate binding. Figure 3 shows the mean solvent accessibility (ASA) values for the binding and non-binding regions in Procarb40 database. We observe that the most frequent carbohydrate binder TRP has a significantly higher ASA in binding locations compared with non-binding ones. Similar higher ASA for binding regions are also observed for other aliphatic residues ALA, GLY, ILE and LEU. Thus, the hydrophobic residues, which are usually in the buried states, do not apparently participate in sugar binding. In order to bind sugars they are expected to be on the surface, thus facilitating their hydrophobic interactions with carbohydrate atoms of protein-carbohydrate complexes and reveals that polar uncharged and certain hydrophobic residues (e.g. TYR, TRP, ALA, LEU and ILE) seem to have higher mean ASA-values in the binding regions. This result contrasts with similar binding sites analysis on DNA-binding proteins, where ASA of charged residues showed a better discrimination between binding and non-binding regions [28]. Most charged and polar residues do not show any difference in their ASA for binding and non-binding regions, presumably because their probability to be on the surface is higher irrespective of their role in binding. For a quick comparison of role of ASA in binding regions of PLD116 and PDNA62 databases with Procarb40, ratio of mean ASA in binding to non-binding regions of the three databases have been plotted in Figure 4 (see Additional file 1). As discussed above, aliphatic residues ILE, LEU and GLY show the highest ratio for Procarb40, in addition to the most frequent binder TRP. Very low values of CYS and VAL residues are not significant as there are very few binding residue of this type (see Table 2).

### Role of Secondary structure

We tried to explore if certain residues prefer any secondary structure for binding to carbohydrates. Results of these statistics are presented in Additional File 1 as Tables 5-10 and Figures 5a-g. If the number of binding sites is resolved into their secondary structure types, very few binding sites are assigned to each category. This leaves the resulting data to be insufficient for any statistical conclusions. These results are therefore not discussed here, but only provided in Additional File 1 for reference.

### Packing Density

We also tried to find out the difference between the packing density of the binding and non-binding residues and observed that there is no statistically significant difference of packing density between binding and non-binding residues.

### Prediction results

Looking at clear preferences of residues for binding carbohydrates (Figure 1), we sought to develop a prediction method, which could take the predisposition of residues and their sequence environments as an input and thereby identify binding residues from the information of protein-sequence. To do so, sequence environment at each residue level could be represented either as binary 20 bit vectors or by the rows of the matrices depicting evolutionary profiles of residues at each location. Sequence neighbor environment could be added as the corresponding rows of this matrix (called position-specific substitution matrix or PSSM) on either side. Schemes of these representations have been extensively developed for the problem of solvent accessibility and other residue-wise features of proteins [55]. Table 3 summarized the results of predictions obtained in this way, using a leave-one-out method. This method also allows us to compute the standard errors in the prediction scores. Further, prediction performance of sequence-only predictors has been compared with those using PSSMs. The best performance for Procarb40 data set was found to be a modest 61%, indicating that the sequence and evolutionary information do not decisively determine a binding site. This not-so-good prediction performance for Procarb40 is is apparently because carbohydrates are diverse and finding overall general rules for their binding sites in proteins may not be possible with the amount of data we have. We need to have large data with sufficient representation of all types of sugars. To ensure that the low performance is caused by the diversity of sugars, we tried to develop a prediction model for only one type of sugar. We tried many differently classified carbohydrates, but due to further small size of data, could only use galactose binding proteins (GalBind18) data set used by Sujatha et al. (2004) [57] to have a sufficient number of binding sites to model. As expected prediction performance for proteins binding to only one type of sugars, was very much higher than all carbohydrates taken together. Table 3 shows that in GalBind18 carbohydrate binding sites could be predicted with as much as 79% specificity and 63% sensitivity. We speculate that much better prediction methods will be developed when a large number of proteins binding to each type of carbohydrates become available.

#### Single sequence versus evolutionary information

It may be a little surprising to note that PSSM based predictions (55%) were somewhat poorer than single sequences (61%) in Procarb40. However in the case of GalBind18 the situation in reversed. Lower values of prediction in PSSM based methods could be due to two reasons. First of all the number of sequences which gave significant alignments with Procarb40 was roughly 400, which is small and hence the evolutionary information transferred to PSSM may not be enough to improve performance. Secondly, the diversity of Procarb40 may lead to higher conservation scores to some residues and hence there would be many false positive predictions by this (that is why the specificity of PSSM based method was as low as 23%). In the case of GalBind18, the situation is reversed because the carbohydrates are more similar and hence conservation of a residue within them does convey positive information about its binding behaviour. Thus PSSMs do not carry false information to the neural network.

### Comparison with other studies

Although some of the results presented in this work may be obvious to some experienced biologists, yet this work is the first attempt to summarize the sequence and structure features of carbohydrate binding proteins in such a comprehensive way. Previous studies have either focused on a small set of proteins aiming to analyze one or a few types of residues [43-45], or tried to focus either on the structural aspect [e.g. [16,17,26]] or just the sequence aspect of these interactions. This is also the first attempt to use sequence and evolutionary information to predict carbohydrate-binding sites using neural network based approach, which has been proved to be successful in making other sequence-based predictions. Earlier, structure-based methods have been employed to develop empirical rules on patches and other structure descriptors with a somewhat better (65%) accuracy. However, sequence-based methods, employing only sequence information presented in this work are new and will have a much wider application as no structure information will be required for prediction. We expect that this will trigger interest in the prediction of carbohydrate binding sites using machine learning methods and the performance will improve with the availability of more data.

## Conclusion

This analysis of protein-carbohydrate interactions in terms of proteins sequence and solvent accessibility reveals that TRP and ARG residues have the highest overall binding propensity for all types of carbohydrates. Planar side chains of polar residues are also confirmed to have overall high propensity of binding. Mean solvent accessibility of hydrophobic residues has been found to be higher for binding regions, whereas charged residues have almost the same solvent accessibility in binding and non-binding regions. A neural network, trained to use evolutionary information of residues and their neighbors could correctly make prediction of binding or non-binding residues with 69–72% specificity and 55–57% sensitivity.

## Methods

### Definition of a binding site

A binding residue is defined as any amino acid in the protein such that any of its atoms is within a cut-off distance from any atom from the sugar in the protein-carbohydrate complex. We tried to determine the best cut-off distance and found that 3.5 Å distance could best separate the binding residues from non-binding ones in the propensity graphs and also gives the best accuracy figures in neural network based predictors. Thus, all the reported results are based on this distance cut-off unless otherwise stated.

### Data Sets

#### Procarb40

PDB search was performed for protein-carbohydrate complexes with a pair-wise similarity of 50% or less. Only one structure was taken in case there were more than one representative from the same family. For polypeptides, only one chain was selected on the basis of maximum number of binding sites present. FASTA formatted sequences were subsequently formatted using *formatdb *program of the BLAST package. BLASTCLUST program [56] at 30% threshold refined our search to 40 structures (Table 1). We call this database Procarb40.

#### GalBind18

This is a data set of 18 Galactose specific proteins selected for another analysis by Sujatha *et al*. [57].

#### PDNA62

This is the (non redundant) data set of 62 DNA-binding proteins [28].

#### PLD116

This is a non-redundant data set of ligand-binding proteins developed for the current study. To begin with, all the 485 protein-ligand complexes were downloaded from Protein-Ligand Database [58] (v1.3 as on 25/01/06). Redundancy among sequences was first removed by using CD-HIT program from [59] with a threshold of 40% sequence identity. This resulted in 178 clusters. FASTA formatted sequences were subsequently formatted using *formatdb *program of the BLAST package. The redundancy was further removed with a threshold of 30% sequence identity using BLASTCLUST program [56]. A data set was thus created, by retaining only the representative ones such that no two sequences in the resulting data set have more than 30% sequence identity. We call this database PLD116.

#### Other data sets

PDB-ALL (47,189 sequences) is a data set of all protein sequences obtained from NCBI. PIR is the sequence data set (283,177 sequences) of Protein Information Resource at Georgetown University [60]. SWISSPROT is another well-known database of sequences [61]. NCBI-NR is a non-redundant data set of all protein sequences compiled from GeneBank, PIR, SwissProt, PDB and other resources by NCBI [62] were also used in the current work.

### Generation of PSSMs

Target sequences are scanned against the reference data sets to compile a set of alignment profiles or position specific scoring matrices (PSSMs) using Position Specific Iterative BLAST (PSI BLAST) program [63]. Three cycles of PSI-BLAST were run for each protein and the scores were saved as profile matrices (PSSMs). NR database of NCBI, PDBAA (database of all amino acid sequences of proteins in PDB), SWISSPROT and PIR were used for building the profiles. Profiles from NR database of NCBI were used for most of the calculations presented in this work unless otherwise specified.

#### Calculation of amino acid composition, solvent accessibility and secondary structure at binding sites

We collected statistics on amino acid residues, which were involved in carbohydrate binding. An attempt was then made to determine whether there was a preference for any particular amino acid residue. Frequency of occurrence for each residue type is calculated and corresponds to the relative number of residues of that type out of all the residues that were found in the carbohydrate-binding proteins.

Solvent accessibility or accessible surface area (ASA) values of Procarb40, PDNA62 and PLD116 complexes were obtained from our earlier database of (relative) solvent accessibility of proteins ASAVIEW [64], whereas the secondary structure was obtained using DSSP program [65].

#### Propensity scores

Propensity of a residue in the binding site was calculated by the formula: -

NBiNiNBallNall
 MathType@MTEF@5@5@+=feaafiart1ev1aaatCvAUfKttLearuWrP9MDH5MBPbIqV92AaeXatLxBI9gBaebbnrfifHhDYfgasaacH8akY=wiFfYdH8Gipec8Eeeu0xXdbba9frFj0=OqFfea0dXdd9vqai=hGuQ8kuc9pgc9s8qqaq=dirpe0xb9q8qiLsFr0=vr0=vr0dc8meaabaqaciaacaGaaeqabaqabeGadaaakeaadaWcaaqaamaaliaabaGaemOta4KaemOqai0aaSbaaSqaaiabdMgaPbqabaaakeaacqWGobGtdaWgaaWcbaGaemyAaKgabeaaaaaakeaadaWccaqaaiabd6eaojabdkeacnaaBaaaleaacqWGHbqycqWGSbaBcqWGSbaBaeqaaaGcbaGaemOta40aaSbaaSqaaiabdggaHjabdYgaSjabdYgaSbqabaaaaaaaaaa@3F2C@

where *NB*_*i *_is the number of residues of type i, which bind to carbohydrate, *N*_*i *_is the total number of residues of type i, NB_all _is the total number of all binding residues, N_all _is the total number of all residues. To compute the propensity score of each residue, the data of binding and non-binding residues were pooled together and a single propensity score was obtained for the entire data of proteins. Also, propensity scores for each protein were calculated separately and standard deviation in all propensity scores for the same residue type was used as the error bar.

### Neural network

#### Neural network inputs

Conservation scores in 20 amino acid positions for every residues form 20 columns (column 3 onwards) of corresponding row in a PSI-BLAST PSSM. For every residue, we make a binary (1 for binding and 0 for non-binding) prediction of that residue being a binding site or not. Input for every prediction is the PSSM score on the row corresponding to this target residue and one more rows on either side (20 × 3 = 60 inputs) as well as two more rows on either side (20 × 5 = 100 inputs).

#### Network architecture and transfer function

A fully connected, feed-forward neural network was constructed using Stuttgart Neural Network Simulator (SNNS) version 4.2, developed at University of Stuttgart [66]. After varying the number of units, and hidden layers, it was found that a network with two units in the hidden layer and a single output unit performed slightly better than other choices.

#### Training and validation

Different datasets and their cross validation were tried. Out of these results are presented for which prediction performance was better than others. We use a leave-one-out approach for training and validation. In this approach, data corresponding to one protein is removed from the data set and the remaining proteins are trained using a neural network. The performance on the left out protein is than measured. The process is systematically repeated for all proteins, leaving them out one by one and measuring their prediction accuracy. Finally reported accuracy scores correspond to the averages of the left out proteins.

Most other procedures for training and assessment of prediction accuracy were the same as in our earlier work [67].

### Assessment of prediction performance

Three scores were used for the measure of prediction performance viz. Sensitivity (S1), Specificity (S2) and their average Net Prediction (NP). They are defined as follows:

Sensitivity (S1)= TP/(TP+FN)

Specificity (S2) = TN/(TN+FP)

Where *TP *stands for correctly identified binding sites, *TN *stands for correctly identified non-binding residues, *FP *stands for number of non-binding residues wrongly identified as binding by predictor, and *FN *is the number of binding residues predicted as non-binding.

## Authors' contributions

This work is part of the doctoral research of AM. SA conceived the problem and designed the protocols. All calculations were performed by AM under SA's guidance and supervision.

## Supplementary Material

Additional file 1 Supplementary Material. The data provides tables and figures with additional information on topics presented in the main text. Some of these results may not be statistically significant due to small size of data. Click here for file

## Figures and Tables

**Table 1:** Showing Procarb40 dataset. (Some cells are left empty as no Pfam ID could be found for them).

**PDB ID**	**Pfam ID**	**Ligand name**	**Ligand formula**	**Ligand ID**
1A7C	Serpins	ALPHA-D-MANNOSE N-ACETYL-D-GALACTOSAMINE N-METHYLCARBONYLTHREONINE	C6 H12 O6 3(C8 H15 N1 O6) 2(C6 H11 N1 O4)	MAN NGA THC
1AU1	Interferon	ZINC ION FUCOSE GLUCOSE D-GALACTOSE ALPHA-D-MANNOSE	ZN1 2+ 2(C6 H12 O6) 4(C6 H12 O6) C6 H12 O6 2(C1 H12 O6)	ZN FUC GLC GAL MAN
1AXM	Fibroblast Growth Factors	SELENOMETHIONINE O2-SULFO-GLUCURONIC ACID N,O6-DISULFO-GLUCOSAMINE	6(C5 H11 N1 O2 SE1) 7(C6 H10 O10 S1) 8(C6 H13 N1 O11 S2)	MSE IDS SGN
1CVN	Lectin legB	CALCIUM ION MANGANESE (II) ION ALPHA-D-MANNOSE	4(CA1 2+) 4(MN1 2+) 12(C1 H12 O6)	CA MN MAN
1E6N	CBM_5_12 Glycol_hydro_18	GLYCEROL SULFATE ION N-ACETYL-D-GLUCOSAMINE	C3 H8 O3 12(O4 S1 2-) 10(C8 H15 N1 O6)	GOL SO4 NAG
1FV3	Toxin_R_bind_C Toxin_R_bind_N Toxin_trans	GLUCOSE PHOSPHATE ION D-GALACTOSE ETHYL-TRIMETHYL-SILANE N-ACETYL-D-GALACTOSAMINE 5-N-ACETYL-BETA-D-NEURAMINIC ACID 5-N-ACETYL-ALPHA-D-NEURAMINIC ACID	2(C6 H12 O6) O4 P1 3- 4(C6 H12 O6) 2(C5 H14 SI1) 2(C8 H15 N1 O6) 2(C11 H19 N1 O9) 4(C11 H19 N1 O9)	GLC PO4 GAL CEQ NGA SLB NAN
1FWU	Ricin_B_lectin	FUCOSE O3-SULFONYLGALACTOSE ALPHA-METHYL-N-ACETYL-D-GLUCOSAMINE	C6 H12 O5 C6 H12 O9 S1 C9 H17 N1 O6	FUC SGA MAG
1G1T	EGF Lectin C	FUCOSE CALCIUM ION D-GALACTOSE O-SIALIC ACID N-ACETYL-O-METHYL-D-GLUCOSAMINE	C6 H12 O5 CA1 2+ C6 H12 O6 C11 H19 N1 O9 C9 H17 N1 O6	FUC CA GAL SIA 1NA
1G5N	Annexin	CALCIUM ION N,O6-DISULFO-GLUCOSAMINE 1,4-DIDEOXY-O2-SULFO-GLUCURONIC ACID 1,4-DIDEOXY-5-DEHYDRO-O2-SULFO-GLUCURONIC ACID	9(CA1 2+) 4(C6 H13 N1 O11 S2) 2(C6 H10 O8 S1) 2(C6 H8 O8 S1)	CA SGN IDU UAP
1GMN	Kringle PAN	O2-SULFO-GLUCURONIC ACID N,O6-DISULFO-GLUCOSAMINE 4-(2-HYDROXYETHYL)-1-PIPERAZINE ETHANESULFONIC ACID	3(C6 H10 O10 S1) 2(C6 H13 N1 O11 S2) 2(C8 H18 N2 O4 S1)	IDS SGN EPE
1GUI	CBM4/9	CALCIUM ION GLYCEROL BETA-D-GLUCOSE	CA1 2+ 5(C3 H8 O3) 6(C6 H12 O6)	CA GOL BGC
1GWM	Family 29 carbohydrate binding module	GLUCOSE COBALT (II) ION 1,2-ETHANEDIOL BETA-D-GLUCOSE	C6 H14 O6 CO1 2+ 8(C2 H6 O2) 5(C6 H12 O6)	GLC CO EDO BGC
1IW6	Bac_rhodopsin	GLUCOSE RETINAL D-GALACTOSE ALPHA-D-MANNOSE 2,3-DI-PHYTANYL-GLYCEROL 2,3-DI-O-PHYTANLY-3-SN-GLYCERO-1-PHOSPHORYL-3'-SN-GLYCEROL-1'-PHOSPHATE	C6 H12 O6 C20 H28 O1 C6 H12 O6 C6 H12 O6 C43 H88 O3 4(C46 H94 O11 P2 2-)	GLC RET GAL MAN L2P L3P
1J8R	PapG _N	GLUCOSE D-GALACTOSE N-ACETYL-D-GLUCOSAMINE SELENOMETHIONINE	C6 H12 O6 2(C6 H12 O6) C8 H15 N1 O6 3(C5 H11 N1 O2 SE1)	GLC GAL NAG MSE
1JPC	B_lectin D-mannose binding lectin	ALPHA-D-MANNOSE	8(C1 H12 O6)	MAN
1LGB	Lectin_legB Transferrin	FUCOSE CALCIUM ION D-GALACTOSE MANGANESE (II) ION ALPHA-D-MANNOSE N-ACETYL-D-GLUCOSAMINE	C6 H12 O6 CA1 2+ C6 H12 O6 MN1 2+ 3(C1 H12 O6) 4(C8 H15 N1 O6)	FUC CA GAL MN MAN NAG
1M5J		ALPHA-D-MANNOSE O1-PENTYL-MANNOSE 2- [N-CYCLOHEXYLAMINO]ETHANE SULFONIC ACID	8(C6 H12 O6) C11 H22 O6 C8 H17 N1 O3 S1	MAN OPM NHE
1OH4		CALCIUM ION GLYCEROL SULFATE ION BETA-D-MANNOSE ALPHA D-GALACTOSE	CA1 2+ 2(C3 H8 O3) O4 S1 2- 5(C6 H12 O6) 2(C6 H12 O6)	CA GOL SO4 BMA GLA
1Q8V	Lectin_legB	CALCIUM ION MANGANESE (II) ION ALPHA-D-MANNOSE PYROGLUTAMIC ACID	2(CA1 2+) 2(MN1 2+) 5(C6 H12 O6) 2(C5 H7 N1 O3)	CA MN MAN PCA
1QFO	V-set	GLUCOSE D-GALACTOSE O-SIALIC ACID	2(C6 H12 O6) 2(C6 H12 O6) 3(C11 H19 N1 O9)	GLC GAL SIA
1RID	Sushi	O2-SULFO-GLUCURONIC ACID N,O6-DISULFO-GLUCOSAMINE	8(C6 H10 O10 S1) 8(C6 H13 N1 O11 S2)	IDS SGN
1SE3	Stap_Strp_tox_C Stap_Strp_toxin	GLUCOSE D-GALACTOSE O-SIALIC ACID	C6 H12 O6 C6 H12 O6 C11 H19 N1 O9	GLC GAL SIA
1SL4	Lectin_C	CALCIUM ION ALPHA-D-MANNOSE	3(CA1 2+) 4(C6 H12 O6)	CA MAN
1SLC	Gal-bind_lectin	D-GALACTOSE ALPHA-D-MANNOSE N-ACETYL-D-GLUCOSAMINE	4(C6 H12 O6) 6(C1 H12 O6) 6(C8 H15 N1 O6)	GAL MAN NAG
1T0W	Chitin_bind_1	AMINO GROUP N-ACETYL-D-GLUCOSAMINE	H2 N1 3(C8 H15 N1 O6)	NH2 NAG
1T8U	Sulfotransfer_1	SODIUM ION SULFATE ION O2-SULFO-GLUCURONIC ACID N,O6-DISULFO-GLUCOSAMINE ADENOSINE-3'-5'-DIPHOSPHATE 1,4-DIDEOXY-5-DEHYDRO-O2-SULFO-GLUCURONIC ACID	2(NA1 1+) O4 S1 2- C6 H10 O10 S1 2(C6 H13 N1 O11 S2) 2(C10 H15 N5 O10 P2) C6 H8 O8 S1	NA SO4 IDS SGN A3P UAP
1ULE		D-GALACTOSE N-ACETYL-D-GLUCOSAMINE	4(C6 H12 O6) 2(C8 H15 N1 O6)	GAL NAG
1UX7	CBM_6	CALCIUM ION SULFATE ION BETA-D-XYLOPYRANOSE	2(CA1 2+) O4 S1 2- 3(C5 H10 O5)	CA SO4 XYP
1UY4		SODIUM ION CALCIUM ION GLYCEROL BETA-D-XYLOPYRANOSE	NA1 1+ CA1 2+ C3 H8 O3 4(C5 H10 O5)	NA CA GOL XYP
1UYY	CBM_6	CALCIUM ION BETA-D-GLUCOSE	4(CA1 2+) 7(C6 H12 O6)	CA BGC
1VBO		ALPHA-D-MANNOSE N-ACETYLALANINE	20(C6 H12 O6) 8(C5 H9 N1 O3)	MAN AYA
1VPS	Polyoma Coat	D-GALACTOSE O-SIALIC ACID N-ACETYL-D-GLUCOSAMINE	5(C6 H12 O6) 10(C11 H19 N1 O9) 5(C8 H15 N1 O6)	GAL SIA NAG
1W9T	CBM_6	SODIUM ION XYLOPYRANOSE BETA-D-XYLOPYRANOSE	6(NA1 1+) 2(C5 H10 O5) 8(C5 H10 O5)	NA XYS XYP
1XT3	Toxin_1	CITRIC ACID N,O6-DISULFO-GLUCOSAMINE 1,4-DIDEOXY-O2-SULFO-GLUCURONIC ACID	C6 H8 O7 3(C6 H13 N1 O11 S2) 3(C6 H10 O8 S1)	CIT SGN IDU
2BOS	SLT beta	BUTYL GROUP GLUCOSE D-GALACTOSE	3(C4 H9) 5(C6 H12 O6) 14(C6 H12 O6)	BUT GLC GAL
2FCP	Plug TonB_dep_Rec	GLUCOSE PHOSPHATE ION D-GALACTOSE NICKEL (II) ION 3-OXO-BUTYRIC ACID 3-OXO-PENTADECANOIC ACID GLUCOSAMINE 1-PHOSPHATE GLUCOSAMINE 4-PHOSPHATE ETHANOL AMINE PYROPHOSPHATE L-GLYCERO-D-MANNO-HEPTOPYRANOSE 3-DEOXY-D-MANNO-OCT-2-ULOSONIC ACID 2-TRIDECANOYLOXY-PENTADECANOIC ACID	C6 H12 O6 O4 P1 3- 2(C6 H12 O6) 2(NI1 2+) C4 H6 O3 C15 H28 O3 C6 H14 N1 O8 P1 C6 H14 N1 O8 P1 C2 H9 N1 O7 P2 2(C7 H14 O7) 2(C8 H14 O8) 2(C28 H54 O4)	GLC PO4 GAL NI LIN LIM GP1 GP4 EA2 GMH KDO LIL
2MPR	LamB	GLUCOSE CALCIUM ION	C_6 _H_12 _O_6 _ Ca ^2+^	GLC CA
3CHB	Enterotoxin b	GLUCOSE D-GALACTOSE O-SIALIC ACID N-ACETYL-D-GALACTOSAMINE N-(EHTYLSULFITE)MORPHOLINE	5(C6 H12 O6) 10(C6 H12 O6) 5(C11 H19 N1 O9) 5(C8 H15 N1 O6) 2(C6 H14 N1 O4 S1)	GLC GAL SIA NGA MES
3MAN	Cellulase	ALPHA-D-MANNOSE	3(C6 H12 O6)	MAN
3MBP		GLUCOSE	3(C6 H12 O6)	GLC

**Table 2:** Propensities of Procarb40, PDNA62 & PLD116 along with their binding and non-binding data

	**PROCARB40**			**PDNA62**			**PLD116**		
**Residue**	**Propensity**	**BS**	**NBS**	**Propensity**	**BS**	**NBS**	**Propensity**	**BS**	**NBS**

**A**	0.43	9	494	0.64	42	389	0.79	109	2684
**C**	0.00	0	29	0.34	7	143	1.07	24	436
**D**	1.41	27	433	0.36	18	292	0.79	84	2009
**E**	1.81	29	356	0.39	32	510	0.92	92	1952
**F**	0.66	9	318	0.77	33	245	1.09	70	1346
**G**	0.80	20	581	0.71	46	372	1.26	176	2633
**H**	1.58	8	114	1.08	39	194	2.09	81	712
**I**	0.12	2	392	0.48	30	373	0.72	70	1837
**K**	1.40	26	419	1.95	180	423	0.59	65	2053
**L**	0.34	8	561	0.38	39	624	0.81	120	2872
**M**	0.19	1	124	0.54	14	149	1.11	42	716
**N**	1.96	38	429	1.45	74	260	1.17	92	1485
**P**	0.40	5	297	0.66	35	307	0.45	38	1597
**Q**	1.54	18	263	1.19	61	272	0.74	46	1123
**R**	2.77	32	246	2.41	208	360	1.80	139	1450
**S**	0.43	9	499	1.33	91	355	1.03	112	2049
**T**	0.70	15	499	1.36	85	325	0.87	90	2030
**V**	0.00	0	472	0.59	40	399	0.73	92	2315
**W**	3.31	23	144	1.40	22	81	2.30	67	518
**Y**	1.68	25	333	1.19	43	189	1.88	125	1189

**Table 3:** Comparison of Binary and PSSM prediction results using jackknife leave-one-out method (binding sites were labeled at 3.5 Å cut-off distance between carbohydrate and protein atoms).

**Data type**	**Validation type**	**Average-sensitivity**	**Average-specificity**	**Average -net Prediction**	**P-value**
GalBind18	Leave1 out (Using PSSM)	0.63 **(0.19)**	0.79 **(0.09)**	0.71 **(0.09)**	0.08859
GalBind18	Leave1 out (Using single sequences)	0.62 **(0.26**)	0.68 **(0.12)**	0.65 **(0.11)**	
Procarb40	Leave1 out (Using PSSM)	0.87 **(0.12)**	0.23 **(0.08)**	0.55 **(0.06)**	0.00209
Procarb40	Leave1 out (Using single sequences)	0.68 **(0.22)**	0.55 **(0.16)**	0.61 **(0.12)**	

Due to a large number of iterations required in a leave-one-out method, the prediction performance has a significant standard deviation, which has been shown in brackets. P-values are for two-tailed t-test conducted to distinguish between the predictions performances of single sequences versus evolutionary information coded by PSSM. In Procarb40, evolutionary profiles give a significantly poorer result than single sequences, due to a high false positive rate (low specificity).
