# Supplementary material for: Sequence and structural features of carbohydrate binding in proteins and assessment of predictability using a neural network
Source: BMC Struct Biol. 2007 Jan 3;7:1. doi: 10.1186/1472-6807-7-1 (PMC1780050; doi:10.1186/1472-6807-7-1)
Supplement: Additional file 1 — Supplementary Material. The data provides tables and figures with additional information on topics presented in the main text. Some of these results may not be statistically significant due to small size of data. [file 1472-6807-7-1-S1.doc]

**SUPPLEMENTARY MATERIAL**

Table 3: Mean ASA between Procarb40 binding and non-bonding sites along-with their p-values.

| **RESIDUE** | **Mean-Bind** | **Mean-NBind** | **Std-Bind** | **Std-NBind** | **p-value** |
| --- | --- | --- | --- | --- | --- |
| **A** | 42.45 | 22.9 | 40.52 | 26.5 | 0.03 |
| **D** | 37.11 | 42.8 | 28.08 | 29.38 | 0.33 |
| **E** | 26.62 | 41.77 | 18.87 | 26.49 | 0.00 |
| **F** | 20.19 | 14.86 | 20.35 | 20.36 | 0.44 |
| **G** | 49.89 | 30.9 | 30.71 | 29.94 | 0.00 |
| **H** | 31.94 | 27.85 | 35.61 | 23.12 | 0.64 |
| **I** | 35.15 | 11.54 | 0.77 | 17.22 | 0.05 |
| **K** | 45.65 | 44.08 | 18.49 | 22.09 | 0.72 |
| **L** | 49.35 | 12.78 | 30.7 | 18.1 | 0.00 |
| **M** | 8.5 | 13.22 | 0 | 19.58 | 0.00 |
| **N** | 38.15 | 41.31 | 30.93 | 29.88 | 0.53 |
| **P** | 35.8 | 33.23 | 25.34 | 24.87 | 0.82 |
| **Q** | 31.73 | 36.89 | 22.17 | 26.9 | 0.43 |
| **R** | 31.07 | 33.84 | 19.33 | 23.61 | 0.52 |
| **S** | 37.85 | 32.33 | 35.57 | 27.85 | 0.56 |
| **T** | 39.45 | 31.09 | 27.06 | 26.33 | 0.23 |
| **W** | 37.95 | 19.17 | 16.63 | 22.06 | 0.00 |
| **Y** | 32.68 | 22.28 | 20.72 | 20.64 | 0.02 |

**Table 4: Ratio of Helix for Procarb40, PDNA62 & PLD116 along with their binding and non-binding data**

|  | **PROCARB40** | | | **PDNA62** | | | **PLD116** | | |
| --- | --- | --- | --- | --- | --- | --- | --- | --- | --- |
| **Residue** | Ratio-H | **BS** | **NBS** | **Ratio-H** | **BS** | **NBS** | **Ratio-H** | **BS** | **NBS** |
| **A** | 0.81 | 1 | 140 | 0.85 | 19 | 212 | 0.88 | 30 | 1225 |
| **C** | - | 0 | 5 | 3.34 | 6 | 29 | 1.60 | 7 | 111 |
| **D** | 0.00 | 0 | 56 | 1.52 | 10 | 108 | 0.83 | 13 | 556 |
| **E** | 0.62 | 2 | 85 | 0.95 | 16 | 267 | 0.97 | 28 | 880 |
| **F** | 4.19 | 3 | 50 | 0.47 | 5 | 86 | 0.82 | 14 | 440 |
| **G** | 1.70 | 1 | 35 | 1.07 | 7 | 53 | 1.49 | 26 | 374 |
| **H** | 0.00 | 0 | 20 | 1.07 | 17 | 79 | 0.71 | 11 | 205 |
| **I** | 0.00 | 0 | 78 | 0.68 | 9 | 170 | 0.89 | 15 | 660 |
| **K** | 0.93 | 2 | 73 | 0.84 | 66 | 196 | 0.67 | 10 | 728 |
| **L** | 0.00 | 0 | 149 | 0.90 | 18 | 331 | 0.90 | 31 | 1209 |
| **M** | 0.00 | 0 | 27 | 0.51 | 3 | 68 | 1.00 | 12 | 301 |
| **N** | 0.61 | 1 | 40 | 1.30 | 32 | 79 | 0.83 | 12 | 344 |
| **P** | 4.56 | 1 | 26 | 1.02 | 4 | 35 | 2.30 | 7 | 190 |
| **Q** | 0.54 | 1 | 58 | 0.94 | 28 | 136 | 1.23 | 16 | 493 |
| **R** | 1.36 | 4 | 48 | 1.02 | 101 | 169 | 1.11 | 41 | 552 |
| **S** | 4.43 | 2 | 50 | 1.32 | 38 | 103 | 1.00 | 19 | 513 |
| **T** | 1.07 | 1 | 64 | 1.07 | 31 | 109 | 1.66 | 25 | 475 |
| **V** | - | 0 | 73 | 0.71 | 9 | 133 | 1.45 | 26 | 682 |
| **W** | 0.74 | 1 | 19 | 0.95 | 11 | 43 | 0.83 | 12 | 169 |
| **Y** | 1.95 | 4 | 56 | 1.05 | 16 | 68 | 0.84 | 21 | 363 |

**Table 5: Ratio of Extended Strand for Procarb40, PDNA62 & PLD116 along with their binding and non-binding data**

|  | **PROCARB40** |  |  | **PDNA62** |  |  | **PLD116** |  |  |
| --- | --- | --- | --- | --- | --- | --- | --- | --- | --- |
| **Residue** | **Ratio-E** | **BS** | **NBS** | **Ratio-E** | **BS** | **NBS** | **Ratio-E** | **BS** | **NBS** |
| **A** | 0.77 | 2 | 158 | 0.96 | 4 | 50 | 1.21 | 26 | 457 |
| **C** | - | 0 | 13 | 1.02 | 1 | 23 | 0.61 | 5 | 129 |
| **D** | 0.98 | 6 | 108 | 0.00 | 0 | 28 | 1.49 | 16 | 223 |
| **E** | 1.58 | 14 | 115 | 0.35 | 1 | 60 | 1.59 | 26 | 290 |
| **F** | 0.46 | 2 | 171 | 1.53 | 13 | 78 | 0.79 | 21 | 409 |
| **G** | 0.83 | 4 | 155 | 0.72 | 3 | 45 | 0.92 | 26 | 372 |
| **H** | 1.56 | 4 | 39 | 1.05 | 4 | 25 | 1.03 | 22 | 160 |
| **I** | 0.00 | 0 | 216 | 1.58 | 11 | 107 | 0.83 | 24 | 691 |
| **K** | 1.59 | 11 | 119 | 1.48 | 26 | 48 | 1.22 | 15 | 355 |
| **L** | 0.34 | 1 | 231 | 0.59 | 3 | 108 | 1.07 | 35 | 684 |
| **M** | 2.86 | 1 | 47 | 0.56 | 1 | 26 | 0.91 | 11 | 182 |
| **N** | 1.31 | 13 | 121 | 1.97 | 8 | 15 | 1.37 | 19 | 190 |
| **P** | 4.74 | 3 | 39 | 0.00 | 0 | 25 | 0.00 | 0 | 142 |
| **Q** | 1.05 | 6 | 92 | 1.32 | 9 | 38 | 0.83 | 8 | 222 |
| **R** | 1.20 | 14 | 97 | 0.88 | 22 | 63 | 1.07 | 35 | 286 |
| **S** | 0.74 | 2 | 166 | 0.59 | 5 | 47 | 1.02 | 22 | 347 |
| **T** | 1.07 | 6 | 206 | 0.75 | 8 | 57 | 0.63 | 18 | 560 |
| **V** | - | 0 | 283 | 1.00 | 11 | 143 | 0.75 | 29 | 907 |
| **W** | 0.92 | 9 | 69 | 1.22 | 5 | 19 | 1.32 | 27 | 130 |
| **Y** | 0.45 | 5 | 171 | 1.22 | 12 | 56 | 0.94 | 41 | 372 |

**Table 6: Ratio of 3-10 Helix for Procarb40, PDNA62 & PLD116 along with their binding and non-binding data**

|  | **PROCARB40** |  |  | **PDNA62** |  |  | **PLD116** |  |  |
| --- | --- | --- | --- | --- | --- | --- | --- | --- | --- |
| **Residue** | **Ratio-G** | **BS** | **NBS** | **Ratio-G** | **BS** | **NBS** | **Ratio-G** | **BS** | **NBS** |
| **A** | 0.00 | 0 | 13 | 1.79 | 2 | 10 | 0.97 | 5 | 108 |
| **C** | - | 0 | 1 | 0.00 | 0 | 3 | 1.24 | 1 | 12 |
| **D** | 0.67 | 2 | 19 | 0.00 | 0 | 15 | 1.04 | 5 | 100 |
| **E** | 0.00 | 0 | 15 | 1.35 | 1 | 12 | 0.34 | 2 | 109 |
| **F** | 3.00 | 1 | 4 | 1.48 | 1 | 5 | 1.03 | 3 | 43 |
| **G** | 0.00 | 0 | 16 | 0.00 | 0 | 1 | 1.05 | 7 | 85 |
| **H** | 1.05 | 2 | 10 | 0.63 | 1 | 9 | 1.15 | 5 | 31 |
| **I** | 0.00 | 0 | 6 | 0.00 | 0 | 4 | 1.30 | 2 | 35 |
| **K** | 0.00 | 0 | 12 | 0.80 | 3 | 10 | 1.84 | 5 | 75 |
| **L** | 1.83 | 1 | 15 | 0.00 | 0 | 19 | 1.39 | 7 | 101 |
| **M** | 0.00 | 0 | 9 | 12.52 | 1 | 0 | 0.65 | 1 | 23 |
| **N** | 0.60 | 2 | 15 | 1.17 | 1 | 3 | 0.88 | 4 | 63 |
| **P** | 0.00 | 0 | 10 | 1.38 | 2 | 13 | 0.40 | 1 | 95 |
| **Q** | 1.17 | 2 | 9 | 2.85 | 3 | 3 | 0.98 | 2 | 46 |
| **R** | 2.05 | 4 | 3 | 0.26 | 1 | 10 | 1.13 | 7 | 53 |
| **S** | 0.00 | 0 | 22 | 1.02 | 2 | 8 | 0.54 | 4 | 120 |
| **T** | 3.53 | 2 | 6 | 0.91 | 2 | 9 | 0.75 | 2 | 51 |
| **V** | - | 0 | 6 | 5.78 | 3 | 3 | 1.29 | 3 | 52 |
| **W** | 2.00 | 4 | 2 | 0.00 | 0 | 2 | 1.39 | 7 | 31 |
| **Y** | 1.18 | 3 | 12 | 1.43 | 2 | 6 | 1.05 | 5 | 39 |

**Table 7: Ratio of Hydrogen-bonded Turn for Procarb40, PDNA62 & PLD116 along with their binding and non-binding data**

|  | **PROCARB40** |  |  | **PDNA62** |  |  | **PLD116** |  |  |
| --- | --- | --- | --- | --- | --- | --- | --- | --- | --- |
| **Residue** | **Ratio-T** | **BS** | **NBS** | **Ratio-T** | **BS** | **NBS** | **Ratio-T** | **BS** | **NBS** |
| **A** | 1.88 | 2 | 61 | 0.89 | 2 | 26 | 1.25 | 10 | 255 |
| **C** | - | 0 | 1 | 0.00 | 0 | 15 | 1.28 | 2 | 36 |
| **D** | 1.58 | 7 | 73 | 2.06 | 4 | 38 | 0.77 | 8 | 335 |
| **E** | 0.90 | 3 | 44 | 1.24 | 3 | 46 | 0.71 | 6 | 235 |
| **F** | 0.00 | 0 | 15 | 0.93 | 2 | 20 | 1.26 | 6 | 108 |
| **G** | 1.17 | 6 | 157 | 0.65 | 7 | 113 | 0.54 | 20 | 756 |
| **H** | 2.94 | 2 | 9 | 1.07 | 5 | 29 | 1.04 | 9 | 100 |
| **I** | 0.00 | 0 | 19 | 0.00 | 0 | 13 | 3.17 | 6 | 63 |
| **K** | 0.26 | 1 | 69 | 1.01 | 15 | 45 | 0.99 | 7 | 308 |
| **L** | 0.00 | 0 | 32 | 2.19 | 5 | 43 | 1.69 | 12 | 218 |
| **M** | 0.00 | 0 | 5 | 1.04 | 1 | 13 | 0.50 | 1 | 46 |
| **N** | 0.62 | 4 | 80 | 0.70 | 8 | 54 | 0.77 | 11 | 309 |
| **P** | 0.00 | 0 | 56 | 0.69 | 3 | 49 | 0.95 | 5 | 302 |
| **Q** | 1.55 | 3 | 29 | 0.47 | 2 | 26 | 4.79 | 5 | 32 |
| **R** | 0.31 | 1 | 29 | 1.14 | 15 | 28 | 1.07 | 11 | 139 |
| **S** | 0.00 | 0 | 71 | 0.79 | 6 | 39 | 0.74 | 9 | 298 |
| **T** | 1.40 | 2 | 50 | 1.63 | 9 | 23 | 0.48 | 3 | 183 |
| **V** | - | 0 | 22 | 2.51 | 3 | 13 | 1.75 | 6 | 116 |
| **W** | 1.03 | 2 | 13 | 0.00 | 0 | 1 | 1.56 | 7 | 44 |
| **Y** | 1.32 | 2 | 21 | 1.56 | 4 | 13 | 1.81 | 14 | 94 |

**Table 8: Ratio of Bend for Procarb40, PDNA62 & PLD116 along with their binding and non-binding data**

|  | **PROCARB40** | | | **PDNA62** |  |  | **PLD116** |  |  |
| --- | --- | --- | --- | --- | --- | --- | --- | --- | --- |
| **Residue** | **Ratio-S** | **BS** | **NBS** | **Ratio-S** | **BS** | **NBS** | **Ratio-S** | **BS** | **NBS** |
| **A** | 1.60 | 1 | 29 | 1.58 | 7 | 29 | 1.00 | 11 | 185 |
| **C** | - | 0 | 2 | 0.00 | 0 | 7 | 1.35 | 3 | 26 |
| **D** | 0.72 | 3 | 58 | 0.39 | 1 | 35 | 1.21 | 18 | 246 |
| **E** | 0.53 | 2 | 41 | 1.86 | 6 | 37 | 0.92 | 10 | 157 |
| **F** | 1.56 | 1 | 19 | 0.00 | 0 | 15 | 0.99 | 7 | 84 |
| **G** | 1.16 | 5 | 106 | 0.89 | 9 | 64 | 0.98 | 46 | 475 |
| **H** | 0.00 | 0 | 9 | 1.67 | 7 | 13 | 1.35 | 15 | 60 |
| **I** | 0.00 | 0 | 14 | 1.52 | 3 | 18 | 1.33 | 7 | 96 |
| **K** | 1.05 | 4 | 52 | 0.85 | 18 | 38 | 0.52 | 5 | 223 |
| **L** | 6.62 | 4 | 33 | 1.42 | 3 | 26 | 1.13 | 11 | 158 |
| **M** | 0.00 | 0 | 12 | 0.95 | 1 | 9 | 1.45 | 4 | 31 |
| **N** | 0.68 | 4 | 58 | 0.64 | 7 | 32 | 1.06 | 17 | 175 |
| **P** | 0.00 | 0 | 32 | 1.25 | 7 | 37 | 1.45 | 8 | 165 |
| **Q** | 0.48 | 1 | 27 | 1.17 | 7 | 19 | 0.73 | 4 | 100 |
| **R** | 0.31 | 1 | 23 | 1.02 | 21 | 23 | 0.45 | 9 | 147 |
| **S** | 0.00 | 0 | 60 | 0.70 | 9 | 41 | 1.12 | 21 | 233 |
| **T** | 1.09 | 2 | 52 | 0.82 | 9 | 33 | 0.52 | 7 | 210 |
| **V** | - | 0 | 22 | 1.30 | 4 | 23 | 1.28 | 10 | 140 |
| **W** | 2.08 | 2 | 4 | 0.00 | 0 | 2 | 0.64 | 4 | 34 |
| **Y** | 2.46 | 4 | 16 | 1.30 | 3 | 7 | 1.18 | 15 | 80 |

**Table 9: Ratio of Coil for Procarb40, PDNA62 & PLD116 along with their binding and non-binding data**

|  | **PROCARB40** | | | **PDNA62** |  |  | **PLD116** |  |  |
| --- | --- | --- | --- | --- | --- | --- | --- | --- | --- |
| **Residue** | **Ratio-C** | **BS** | **NBS** | **Ratio-C** | **BS** | **NBS** | **Ratio-C** | **BS** | **NBS** |
| **A** | 1.29 | 3 | 92 | 1.04 | 8 | 59 | 1.26 | 27 | 428 |
| **C** | - | 0 | 6 | 0.00 | 0 | 65 | 0.65 | 5 | 115 |
| **D** | 0.92 | 9 | 113 | 0.66 | 3 | 66 | 0.80 | 21 | 536 |
| **E** | 1.34 | 8 | 50 | 0.79 | 5 | 85 | 1.08 | 17 | 272 |
| **F** | 1.07 | 2 | 48 | 1.72 | 12 | 38 | 1.03 | 17 | 236 |
| **G** | 0.78 | 4 | 109 | 1.36 | 20 | 94 | 1.08 | 48 | 542 |
| **H** | 0.00 | 0 | 26 | 0.62 | 5 | 36 | 0.73 | 15 | 150 |
| **I** | 5.26 | 2 | 53 | 1.26 | 7 | 56 | 1.19 | 14 | 260 |
| **K** | 1.07 | 8 | 86 | 1.07 | 52 | 85 | 1.65 | 21 | 343 |
| **L** | 1.09 | 2 | 94 | 1.35 | 9 | 89 | 0.99 | 23 | 458 |
| **M** | 0.00 | 0 | 23 | 1.82 | 7 | 32 | 1.37 | 12 | 121 |
| **N** | 1.00 | 14 | 112 | 0.70 | 17 | 75 | 0.99 | 28 | 376 |
| **P** | 0.34 | 1 | 129 | 0.97 | 19 | 145 | 0.92 | 17 | 678 |
| **Q** | 1.12 | 5 | 46 | 0.91 | 12 | 49 | 0.99 | 10 | 219 |
| **R** | 1.06 | 8 | 40 | 1.01 | 48 | 61 | 1.10 | 34 | 255 |
| **S** | 1.58 | 5 | 126 | 0.88 | 31 | 114 | 1.03 | 34 | 500 |
| **T** | 0.43 | 2 | 115 | 0.90 | 26 | 91 | 1.20 | 34 | 509 |
| **V** | - | 0 | 61 | 1.04 | 10 | 80 | 0.92 | 16 | 383 |
| **W** | 0.74 | 5 | 31 | 1.39 | 6 | 11 | 0.54 | 8 | 100 |
| **Y** | 1.21 | 7 | 54 | 0.68 | 6 | 35 | 0.89 | 24 | 217 |

Table 10: P-values for binding site propensities between Procarb40/PDNA62 and Procarb40/PLD116.

| **Residue** | **Procarb40-PDNA62** | **Procarb40-PLD116** |
| --- | --- | --- |
| **A** | 0.44 | 0.05 |
| **C** | 0.21 | 0.16 |
| **D** | 0.01 | 0.02 |
| **E** | 0.01 | 0.18 |
| **F** | 0.95 | 0.08 |
| **G** | 0.25 | 0.28 |
| **H** | 0.29 | 0.39 |
| **I** | 0.09 | 0.02 |
| **K** | 0.29 | 0.01 |
| **L** | 0.39 | 0.00 |
| **M** | 0.04 | 0.01 |
| **N** | 0.13 | 0.10 |
| **P** | 0.10 | 0.43 |
| **Q** | 0.72 | 0.09 |
| **R** | 0.68 | 0.70 |
| **S** | 0.00 | 0.00 |
| **T** | 0.01 | 0.70 |
| **V** | 0.00 | 0.00 |
| **W** | 0.02 | 0.90 |
| **Y** | 0.01 | 0.64 |

Table 11: P-values for binding site propensities at Helix between Procarb40/PDNA62 and Procarb40/PLD116.

| **Residue** | **Procarb40-PDNA62** | **Procarb40-PLD116** |
| --- | --- | --- |
| **A** | 0.93 | 0.60 |
| **C** | - | - |
| **D** | 0.31 | - |
| **E** | 0.59 | 0.34 |
| **F** | 0.00 | 0.42 |
| **G** | 0.27 | 0.84 |
| **H** | - | - |
| **I** | 0.59 | 0.62 |
| **K** | 0.95 | 0.05 |
| **L** | - | - |
| **M** | - | - |
| **N** | 0.50 | 0.88 |
| **P** | 0.93 | 0.82 |
| **Q** | 0.16 | 0.03 |
| **R** | 0.01 | 0.13 |
| **S** | 0.37 | 0.42 |
| **T** | 0.64 | 0.51 |
| **V** | - | - |
| **W** | 0.16 | 0.33 |
| **Y** | 0.03 | 0.01 |

Table 12: P-values for binding site propensities at Beta-Strand between Procarb40/PDNA62 and Procarb40/PLD116.

| **Residue** | **Procarb40-PDNA62** | **Procarb40-PLD116** |
| --- | --- | --- |
| **A** | 0.66 | 0.11 |
| **C** | - | - |
| **D** | - | 0.75 |
| **E** | 0.06 | 1.00 |
| **F** | 0.08 | 0.23 |
| **G** | 0.24 | 0.44 |
| **H** | 0.55 | 0.02 |
| **I** | - | - |
| **K** | 0.17 | 0.05 |
| **L** | 0.91 | 0.06 |
| **M** | - | - |
| **N** | 0.29 | 0.24 |
| **P** | 0.13 | 0.00 |
| **Q** | 0.44 | 0.80 |
| **R** | 0.43 | 0.85 |
| **S** | 0.26 | 0.09 |
| **T** | 0.63 | 0.38 |
| **V** | - | - |
| **W** | 0.10 | 0.88 |
| **Y** | 0.02 | 0.03 |

Table 13: P-values for binding site propensities at Coil between Procarb40/PDNA62 and Procarb40/PLD116.

| **Residue** | **Procarb40-PDNA62** | **Procarb40-PLD116** |
| --- | --- | --- |
| **A** | 0.26 | 0.09 |
| **C** | - | - |
| **D** | 0.01 | 0.10 |
| **E** | 0.03 | 0.69 |
| **F** | 0.23 | 0.45 |
| **G** | 0.85 | 0.17 |
| **H** | 0.47 | 0.32 |
| **I** | 0.97 | 0.68 |
| **K** | 0.16 | 0.77 |
| **L** | 0.16 | 0.12 |
| **M** | - | - |
| **N** | 0.55 | 0.70 |
| **P** | 0.06 | 0.17 |
| **Q** | 0.65 | 0.30 |
| **R** | 0.24 | 0.42 |
| **S** | 0.15 | 0.30 |
| **T** | 0.01 | 0.02 |
| **V** | 0.43 | 0.42 |
| **W** | 0.46 | 0.64 |
| **Y** | 0.87 | 0.83 |

**Figure Legends:**

**Figure 4: Comparison of ratio between Procarb40, PDNA62 and PLD116.**

This figure shows the ratio between mean ASA binding and non-binding sites of Procarb40, PDNA62 and PLD116.

**Figure 5: Normalized propensities (BS propensity/ Structure propensity)**

Comparison of ratio between Procarb40, PDNA62 and PLD116 for Helix 5(a) Extended Strand 5(b) Beta Bridge 6(c) 3-Helix 7(d) Hydrogen-bonded turn 8(e) Bend 9(f) and Coil 10(g).

**Figure 4:**

Figure 5(a-g):

Fig. 5a

**Fig. 5b**

**Fig. 5c**

**Fig. 5d**

**Fig. 5e**

**Fig. 5f**

**Fig. 5g**
